# Fire-Induced Changes in Soil Properties and Bacterial Communities in Rotational Shifting Cultivation Fields in Northern Thailand

**DOI:** 10.3390/biology13060383

**Published:** 2024-05-27

**Authors:** Noppol Arunrat, Chakriya Sansupa, Sukanya Sereenonchai, Ryusuke Hatano, Rattan Lal

**Affiliations:** 1 Faculty of Environment and Resource Studies, Mahidol University, Nakhon Pathom 73170, Thailand; sukanya.ser@mahidol.ac.th; 2 Department of Biology, Faculty of Science, Chiang Mai University, Chiang Mai 50200, Thailand; chakriya.sansupa@gmail.com; 3 Laboratory of Soil Science, Research Faculty of Agriculture, Hokkaido University, Sapporo 060-8589, Japan; hatano@agr.hokudai.ac.jp; 4 CFAES Rattan Lal Center for Carbon Management and Sequestration, The Ohio State University, 2021 Coffey Rd, Columbus, OH 43210, USA; lal.1@osu.edu

**Keywords:** fire, rotational shifting cultivation, soil bacterial community, soil physicochemical properties, terrestrial microbiology

## Abstract

**Simple Summary:**

The dynamics of the soil bacterial communities in rotational shifting cultivation fields were investigated. A six-year interval of fallow years did not result in any differences in the soil bacterial communities. A recovery in the abundance of the soil bacterial communities was observed during the rainy season. An increase in bacterial richness occurred during the year after a burn. After one year, the diversity of the bacterial communities reverted to pre-burning levels.

**Abstract:**

Fire is a common practice in rotational shifting cultivation (RSC), but little is known about the dynamics of bacterial populations and the impact of fire disturbance in northern Thailand. To fill the research gap, this study aims to investigate the dynamics of soil bacterial communities and examine how the soil’s physicochemical properties influence the bacterial communities in RSC fields over a period of one year following a fire. Surface soil samples (0–2 cm depth) were collected from sites with 6 (RSC-6Y) and 12 (RSC-12Y) years of fallow in Chiang Mai Province, northern Thailand at six different time points: before burning, 5 min after burning (summer), 3 months after burning (rainy season), 6 months after burning (rainy season), 9 months after burning (winter), and 12 months after burning (summer). The results revealed a reduction in the soil bacterial communities’ diversity and an increase in soil nutrient levels immediately after the fire. The fire significantly influenced the abundance of Firmicutes, Proteobacteria, Acidobacteria, and Planctomycetes, but not that of Actinobacteria. At the genus level, Bacillus, Conexibacter, and Chthoniobacter showed increased abundance following the fire. During the rainy season, a recovery in the abundance of the soil bacterial communities was observed, although soil nutrient availability declined. Soil physicochemical properties such as pH, organic matter, organic carbon, electrical conductivity, cation exchange capacity, nitrate-nitrogen, available phosphorus, exchangeable potassium, total nitrogen, bulk density, sand, and silt contents significantly influenced the composition of bacterial communities. Alpha diversity indices indicated a decrease in diversity immediately after burning, followed by an increase from the early rainy season until the summer season, indicating that seasonal variation affected the composition of the soil bacterial communities. After one year of burning, an increase in bacterial richness was observed, while the diversity of the bacterial communities reverted to pre-burning levels.

## 1. Introduction

Shifting cultivation is a type of rotational agricultural technique, commonly referred to as slash-and-burn or swidden. Moreover, it is a culture that has been transmitted by local indigenous populations [1]. Shifting cultivation is a relatively common practice, particularly in the highlands of south and southeast Asia [2]. It involves clearing a patch of vegetation using the slash-and-burn method and cultivating assorted crops in the cleared land [3]. The fields are left fallow after harvesting to facilitate the recovery of soil nutrients and promote the growth of plants through secondary succession [4,5]. The plant diversity may completely recover between 20 and 40 years after a fire [6]. In general, 14–15 months after the combustion phase, the soil microbial population can be the same as before [7]. In the mountainous regions of northern Thailand, shifting cultivation has been a long-standing traditional farming practice among hill tribes. However, shifting cultivation areas in Thailand are referred to as rotational shifting cultivation (RSC), because cutting native forests and building new settlements in protected forests are not allowed [3].

Fire is an element of an ecosystem that is utilized to encourage the germination or spread of plant species, especially in areas with deciduous forests [8]. However, wildfires have the apparent disadvantage of being difficult to control. One of the fundamental components of shifting agriculture is fire, which serves as a tool for land preparation [9]. However, burning vegetation can indeed alter the characteristics of the soil and its microbial community, impacting the soil’s texture, structure, fertility, and physicochemical properties [10,11]. Burning of cultivated areas can alter the soil’s characteristics, depending on the fire’s severity and intensity. Low-intensity fires are shown to increase soil organic carbon (SOC), whereas high-intensity fires reduce SOC [12]. Ash deposition from biomass burning increases pH, calcium (Ca), phosphorus (P), potassium (K), magnesium (Mg), and the sum of basic cations [13]. The highest losses of eroded soil carbon (C) and nitrogen (N) were recorded in the first 2 years following severe wildfires in the western USA [14]. Hence, prescribed fires, specifically low-intensity fires, can be used to manage fuel and mitigate negative effects on soil properties [15].

Soil bacteria play a crucial role in biogeochemical cycles, supplying essential nutrients to the soil, and promoting plant growth [16]. Aeromicrobium, Agromyces, and Bacillus are among the common bacteria found in soil free from plant diseases [17]. Bacillus is crucial for plant development [18]. However, fire affects soil bacteria through heat and alteration of soil properties [19]. Smith et al. [20] reported a widespread presence of the Bacillus genus in the boreal forest near Chisholm, Alberta, Canada. In Bangladesh’s Chittagong Hill Tracts, the Cocci, Bacillus, and Streptococcus species were found in soil under shifting cultivation [21]. Arunrat et al. [22] conducted the first study in Thailand to identify soil bacterial communities in a continuous 5-year fallow of a RSC field. They observed that Candidatus Udaeobacter dominated during the summer and winter seasons, while Bacillus was the dominant genus during the rainy season. Arunrat et al. [23] reported that the phylum Firmicutes presented a substantial increase of around 95% after a fire, with Bacillus being dominant. In the fungal community, Ascomycota experienced a significant increase, and Penicillium a dominant increase, after the fire in a RSC field in Thailand.

Hamman et al. [7] reported that the microbial communities in the burnt sites exhibited structural differences compared to the unburnt sites, while microbial biomass did not change after burning. They also indicated temperature, C content, and soil pH are the main factors that influence soil bacterial development. Some prior studies have revealed that the temporal dynamics of microbial communities are primarily influenced by seasonal periodicity [24,25,26], which can be attributed to the complex interactions among climate factors and variations in soil physicochemical properties. However, the dynamics of bacterial populations in shifting agricultural areas and the impact of fire disturbance remain unknown in northern Thailand. To gain a better understanding of these changes, this study was aimed at assessing the dynamics of soil bacterial communities and assessing the influence of soil physicochemical properties on bacterial communities for one year after a fire. This study was designed to test the following hypotheses: (i) burning would result in an immediate reduction in soil bacterial community diversity and an increase in soil nutrient levels, (ii) the abundance of the soil bacterial communities would begin to increase after 3 months of burning due to seasonal changes, while the availability of soil nutrients would decline, and (iii) seasonal variation and soil physicochemical properties would influence the composition of the soil microbial communities. The findings of this study can serve as a valuable scientific reference for understanding the recovery of soil properties and bacterial communities following a fire in RSC fields.

## 2. Materials and Methods

### 2.1. Study Areas

**Figure 1:**
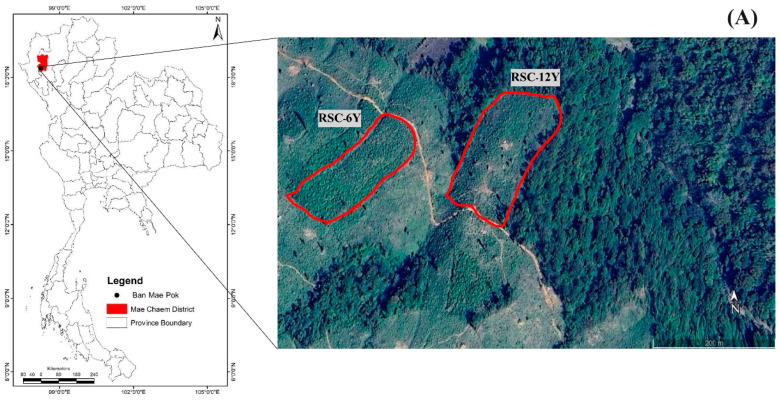

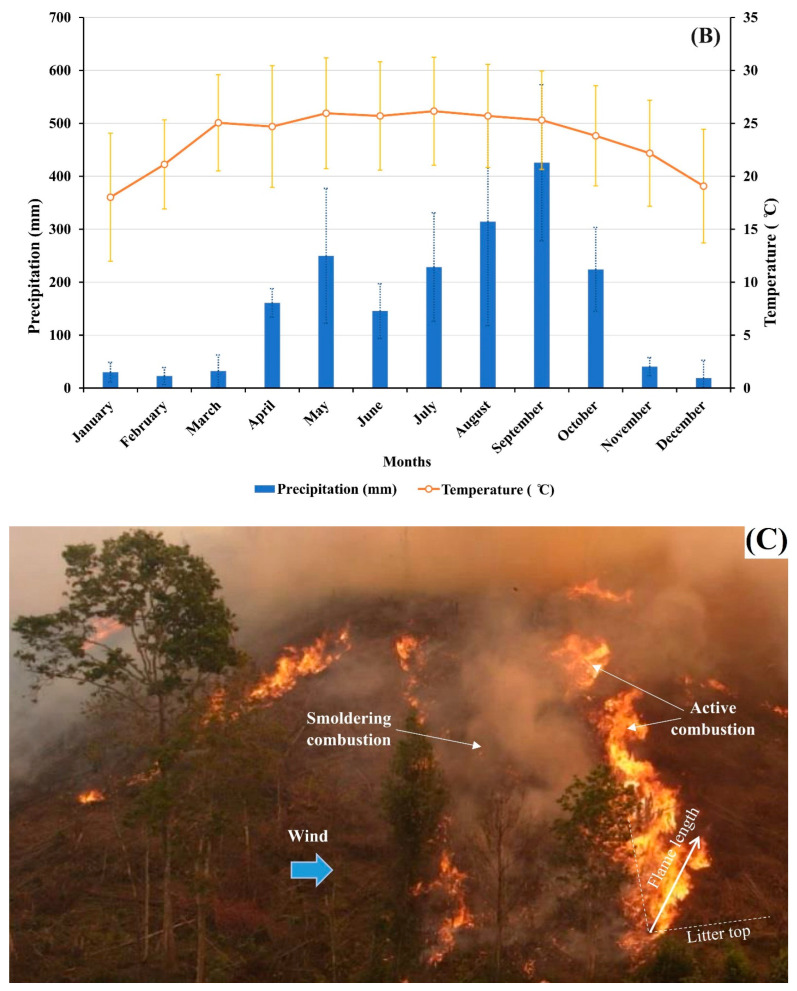

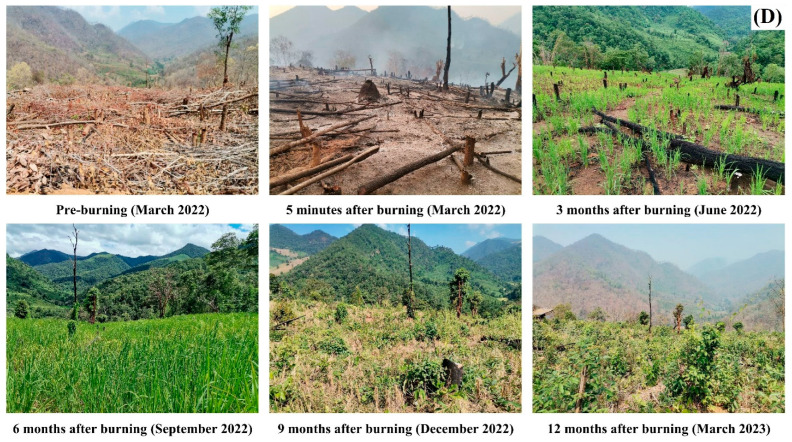
Study area. (**A**) Location of RSC-6Y and RSC-12Y sites, (**B**) temperature and precipitation during 2021–2022, (**C**) fire behaviors, and (**D**) RSC fields over six time points. Photos were taken by Noppol Arunrat.

The study sites were located in Ban Mae Pok, Ban Thab Subdistrict, Mae Chaem District, Chiang Mai Province, northern Thailand (Figure 1A) [27]. Based on the data recorded by the Thai Meteorological Department in Chiang Mai Province (Doi Ang Khang and Mueang Chiang Mai stations) during 2021–2022, the annual rainfall varied between 1105–2688 mm (rainy season: May to October). The winter season (October and February) had minimum temperatures ranging from 3.2–22.1 °C, while the summer season (February to April) reached maximum temperatures ranging from 35–40 °C (Figure 1B). The soils in the highlands of Thailand with a slope greater than 35% were classified as slope complex soil series [28].

Two RSC fields were chosen for their similarity in microclimate and prior land use for the cultivation of upland rice. RSC-12Y (18°23′17′′ N, 98°11′41′′ E; elevation 692 m a.s.l; slope gradient 28%) was left fallow for 12 years after a harvest of upland rice. The field was subsequently cleared, burned, and used for upland rice cultivation in 2022. RSC-6Y (18°23′16′′ N, 98°11′32′′ E; elevation 729 m a.s.l; slope gradient 31%) was left fallow for 6 years after a harvest of upland rice. The RSC-12Y and RSC-6Y fields were cleared, the residues were burned, and the fields were used for upland rice cultivation in 2022 [27].

### 2.2. Experimental Design, Fire Measurements and Soil Sampling

Three sampling lines were established at intervals of 25 m for RSC-12Y and 15 m for RSC-6Y, ensuring statistical independence between lines. The sampling lines, with lengths of 170 m for RSC-12Y and 150 m for RSC-6Y, began at the upper slope and concluded at the lowest slope. Burning was conducted on 30 March 2022 at 15.00–17.00 h. The fire temperature during burning was measured using an infrared thermometer (PONPE 470IR). Fire measurements, including flame length, flame residence time, and spread rate, were conducted using drones for top-view image and video recordings, as well as direct observation by personnel on the ground (Figure 1C). Flame length was measured from the average flame tip to the middle of the flaming zone at the base of the fire [29]. Flame residence time was determined from video recordings, beginning when the flame front reached a specific point along the fuel bed and ending when flaming combustion was completed at that location [29]. The rate of fire spread was calculated using the cumulative spread rate method, which is derived by dividing the total distance traveled by a fire by the total time of travel [30].

In each sampling line of both the RSC-12Y and RSC-6Y fields, soil samples were collected from the surface layer (0–2 cm depth) at three sample plots, each with an approximate area of 1 × 1 m. While collecting soil samples, a measuring tape was used to ensure that samples were taken at precisely 0–2 cm depth. These sample plots were spaced approximately 50 m apart from each other. Soil samples from each plot of each sampling line were composited to obtain one composite sample per sampling line. This approach could reduce the variability in the richness and diversity of soil bacteria along the slopes, which might occur due to variations in soil nutrients caused by erosion processes in RSC fields. Collecting soil samples at a depth of 0–2 cm was considered appropriate for capturing the dynamics of soil bacteria, as this surface layer is more sensitive to changes in environmental conditions [22,31,32,33]. Stones, grasses, roots, ash, charcoal, and residues were removed manually. Steel knives were used to collect soil samples, following which approximately 1 kg of soil was transferred into a plastic bag for the analysis of the soil’s physical and chemical properties. Meanwhile, around 100 g of soil was placed into zip-lock plastic bags and cooled at −20 °C for DNA extraction. Soil sampling was conducted at six different time points: before burning (March 2022), after burning at 5 min (March 2022), 3 months (June 2022), 6 months (September 2022), 9 months (harvest, December 2022), and 12 months (March 2023) (Figure 1D). A total of 36 soil samples were taken from the two RSC fields, comprising six time points. For each plot from where soil samples were obtained, soil temperature and soil moisture content were also recorded at 2 cm depth by using a Type K Thermocouple (PONPE 422 PR) and soil moisture meter (PONPE 301SM), respectively.

### 2.3. Analysis of Soil Physical and Chemical Properties

The soil bulk density (BD) at 0–5 cm was measured using a steel core (5.0 cm diameter and 5.5 cm length) and dried at 105 °C for 24 h [23]. Bulk samples were air-dried, grinded and sieved through a 2-mm sieve. Soil texture was determined on sieved samples by the hydrometer method [23]. Soil pH was measured by a pH meter (1:1 solids in water) [34], electrical conductivity (ECe) by using an EC meter [34], the cation exchange capacity (CEC) by the NH_4_OAc method (pH 7.0) [32], total N (TN) by the micro-Kjeldahl method [34], and ammonium N (NH_4_-N) and nitrate–N (NO_3_-N) by the KCl extraction method [34]. In addition, exchangeable calcium (exch. Ca), magnesium (exch. Mg), and potassium (exch. K) were measured using atomic absorption spectrometry with NH_4_OAc pH 7.0 extraction [35,36]. Available phosphorus (avail. P) was measured using the Bray II extraction method [35,37] and soil organic carbon (SOC) content was determined by the potassium dichromate (K_2_Cr_2_O_7_) in sulfuric acid method [35,38], and converted to SOM by multiplying by 1.724.

### 2.4. DNA Extraction, Bacterial 16s Amplification, and Sequencing

DNA was extracted from the soil using a DNeasy PowerSoil Pro DNA Kit (Qiagen, Hilden, Germany), following the manufacturer’s protocols. The extracted DNA was amplified using primers 341F (5′-CCTAYGG-GDBGCWSCAG) and 805R (5′-GGACTAC-NVGGGTHTCTAAT-3′), which amplified the V3–V4 region of the 16S rRNA gene [39]. The PCR product was subjected to sequencing using the paired-end Illumina Miseq platform at the Omics Sciences and Bioinformatics Center of Chulalongkorn University (Bangkok, Thailand).

### 2.5. Bioinformatics Analysis

Bioinformatics analysis was performed on QIIME2 to identify bacterial taxonomy and estimate the abundance of each taxon [40]. Briefly, raw sequence reads were imported to QIIME2. Forward and reverse primers were cut by cutadapt [41]. The DADA2 plugin was used for quality-filtering, merging, and chimera removal with the following parameters: forward read length = 230 bp, reverse read length = 210 bp, maxEE = 5, and overlap = 12 bp [42]. Amplicon sequence variants (ASVs) with less than two sequence reads (singletons) were removed. Taxonomy was assigned using the Silva v.138 database [43,44] and ASVs that were assigned to mitochondria or chloroplasts were filtered out. The remaining ASVs were then normalized to the smallest number of sequences from each sample using the rarefy plugin. Predictive enzyme abundance was assigned to the dataset using PICRUSt2 [45]. PICRUSt2 predicts potential functions based on reference genes. While this tool provides valuable insights into microbial functions, it is important to note that the results from these predictions still need to be validated by experimental data.

### 2.6. Statistical Analysis

Statistical analyses were performed on PAST [46] and the R program. The soil’s physical and chemical properties at six time points were compared with one-way repeated measures ANOVA and post hoc Tukey’s HSD tests. The alpha diversity indices, including observed richness, ACE, Simpson, and Shannon indices, were computed by the microeco package [47]. The alpha diversity indices in the two study sites and at the 6 sampling times were analyzed by two-way analysis of variance (two-way ANOVA). Differences of the indices between each of the sampling times were tested by ANOVA with repeated measures. Bacterial community composition was analyzed and visualized by non-metric multidimensional scaling (NMDS). The differences between each composition were tested by two-way permutational multivariate analyses of variance (two-way PERMANOVA). A redundancy analysis (RDA) was used to determine the influence of soil properties on soil bacterial community compositions, and the significance of the correlations between soil properties and bacterial communities was confirmed using the Mantel test. Differences between the abundances of bacteria at the phyla and genus levels and predictive enzymes were tested using ANOVA with repeated measures. Spearman’s rank correlation was used to test the correlations between the abundances of genera, predictive enzymes, and soil properties.

## 3. Results

### 3.1. Soil Moisture, Soil Temperature, Fire Behaviors, and Soil Physicochemical Properties

No significant differences in soil moisture content were observed between pre-burning and 5 min after burning, likely due to the short flame residence time (Table 1) resulting in less moisture evaporation. However, significant differences were observed at 3 and 6 months after burning due to high precipitation (Figure 1B). At a depth of 0–2 cm, a significant increase in soil temperature was observed even at 5 min after burning, reaching an average of 46.0 °C and 47.8 °C for the RSC-6Y and RSC-12Y sites, respectively. During burning, the fire temperature in the litter layer ranged from 253 to 612 °C and 315 to 754 °C for the RSC-6Y and RSC-12Y sites, respectively. This is consistent with the fire temperatures in the topsoil of the slash and burn agricultural system in the rural community of Tijuco Preto in the Prudentópolis municipality of southern Brazil, which ranged from 534 to 777 °C [48]. Flame length and flame residence time during burning were measured in the range of 3.6 to 5.5 m and 22.0 to 33.0 s, respectively, while the spread rates varied from 12.5 to 15.5 m/min (Table 1).

Five minutes after burning, the soils exhibited significantly lower levels of SOM, SOC, TN, NO3-N, and clay content. Conversely, burned soils showed higher pH, ECe, NH_4_-N, sand content, and soil nutrients (Avail. P, Exch. K, Exch. Ca, and Exch. Mg). However, bulk density and CEC remained unchanged at 5 min after burning. At 1 year after burning, the soil pH, ECe, SOM, SOC, Avail. P, and NH_4_-N were higher than the initial values (pre-burning), whereas the levels of TN, Exch. K, Exch. Ca, and Exch. Mg declined over time points (Table 2 and Table 3). However, it may be crucial to measure the bulk of plant litter in future studies to enhance our understanding of how soil temperatures impact the soil surface, especially in relation to SOM.

### 3.2. Bacterial Richness and Diversity

**Figure 2:**
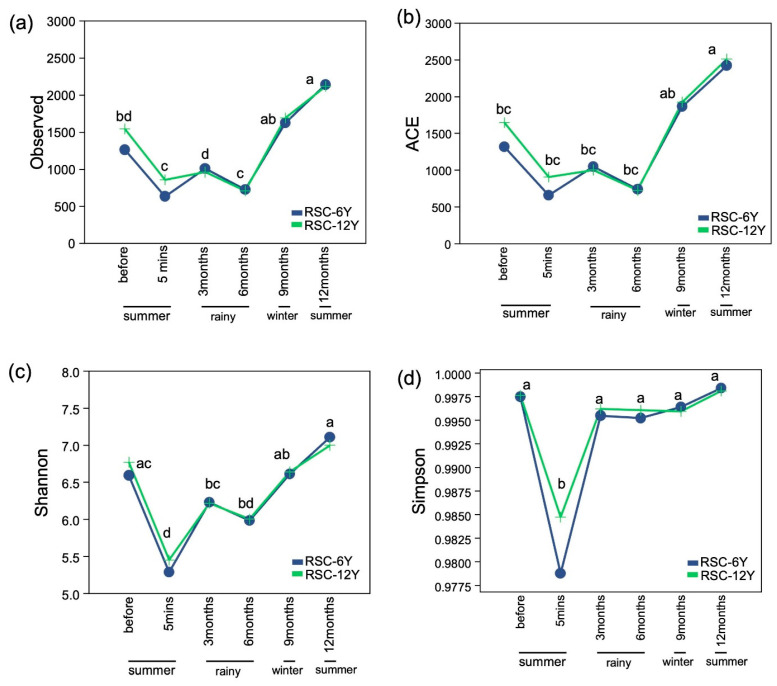
Alpha diversity indices across all samples. (**a**) Observed richness, (**b**) ACE richness, (**c**) Shannon diversity index, and (**d**) Simpson diversity index. Different letters in the plots indicate statistically different (*p* < 0.05).

The bacterial diversity in two rotational shifting cultivations at six time points was examined in this study. A total of 427,788 high-quality sequence reads (11,883 sequences per sample) and 19,297 ASVs were obtained from the sequencing analysis. Alpha diversity, which represents the richness (observed richness and ACE) and diversity of bacteria (Shannon’s and Simpson’s indices), was observed. The two-way ANOVA analysis (Table 4) showed that sampling time had a significant impact on all diversity indices, whereas site and the interaction between both factors did not. Furthermore, the richness and diversity decreased immediately after burning (5 min) and increased three months later. The observed richness and ACE in the wet or rainy season (3 and 6 months) were comparable to those observed 5 min after burning. However, they significantly increased in the dry season (winter and summer, 9 and 12 months after burning) (Figure 2a,b). On the other hand, the Shannon’s and Simpson’s indices increased significantly in the wet or rainy season (3 months after burning). The Shannon’s index was low during the rainy season and increased during the dry season (winter and summer) (Figure 2c). However, no significant changes were observed between the Simpson’s diversity indices for different sampling times after 3 months of burning (Figure 2d).

### 3.3. Bacterial Taxonomic Distribution

**Figure 3:**
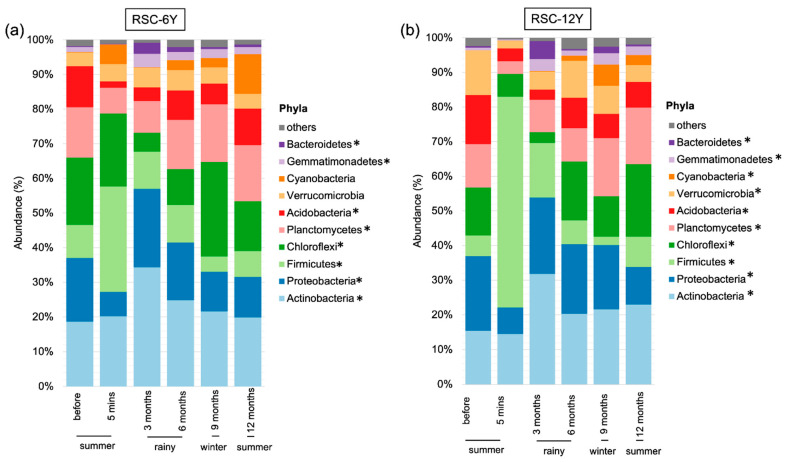
Stacked bar plot showing the most abundant bacterial phyla in (**a**) rotational shifting cultivation—6 years (RSC-6Y) and (**b**) rotational shifting cultivation—12 years (RSC-12Y). * denote statistically significant (*p* < 0.05).

Overall, we observed 35 phyla, 91 classes, 184 orders, 258 families, and 502 genera. The most abundant phyla across all samples belonged to Actinobacteria, Proteobacteria, Firmicutes, Chloroflexi, and Planctomycetes (Figure 3). Burning significantly affected the abundance of several phyla, such as Firmicutes, Proteobacteria, Acidobacteria, and Planctomycetes, while it did not have a significant effect on certain phyla, such as Actinobacteria. The abundance of Actinobacteria at 5 min after burning was similar to that before burning, but increased during the wet or rainy season (3 months after burning) and decreased during the dry season (winter and summer; 9 and 12 months after burning) (Figure 3). At the genus level, the most abundant genera across all samples were *Bacillus,* followed by *Geodermatophilus*. Burning significantly affected the abundance of *Bacillus*, *Conexibacter*, and *Chthoniobacter*. The abundance of each genus changed at each time point. One year after burning, the proportions of bacteria were not similar to those before burning.

#### 3.3.1. Taxonomic Distribution in RSC-6Y

Chloroflexi (19.44%), Actinobacteria (18.61%), and Proteobacteria (18.39%) dominated the community before burning. However, the most fluctuating phyla after burning were Fermicute, Proteobacteria, Acidobacteria, and Planctomycetes. The abundance of Firmicutes increased from 9% to 30% after burning (5 min), then decreased to 10% during the wet or rainy season (3 and 6 months after burning), to 4% during winter (9 months after burning), and 7% in summer (12 months after burning). Proteobacteria and Acidobacteria decreased from 18% to 7% and 12% to 2%, respectively, after burning (5 min). The abundance of these two phyla increased during the rainy season, after 3 months (Proteobacteria: 22% and Acidobacteria: 4%) and 6 months (Proteobacteria: 16% and Acidobacteria: 8%). Then, during summer (12 months after burning), Proteobacteria totaled 11.67% and Acidobacteria totaled 10.53% (Figure 3a).

**Figure 4:**
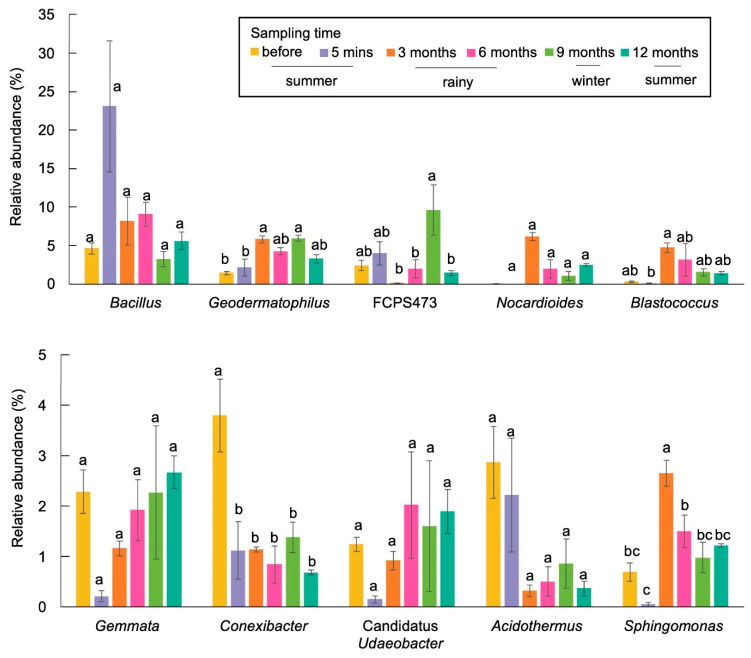
Bar plot showing the abundance of the most abundant genera in RSC-6Y. Different letters in the plots indicate statistically different (*p* < 0.05).

The most abundant genus before burning was *Bacillus* (4.65%), followed by *Conexibacter* (3.80%) and *Gemmata* (2.29%). The abundance of *Bacillus* and *Nocardioides* increased after burning (5 min), while that of *Gemmata*, *Conexibacter*, Candidatus *Udaeobacter*, and *Sphingomonas* decreased. However, significant change was observed only in *Conexibacter*. The data from this study also showed that *Sphingomonas* significantly changed with the changes of the seasons. Their abundance was high during the wet or rainy season (3 and 6 months after burning) and significantly decreased during the dry season (winter and summer) (Figure 4). One year after burning, the most prevalent genera belonged to *Bacillus* (5.60%), followed by *Geodermatophilus* (3.30%) and *Gemmata* (2.67%).

Spearman’s rank analysis revealed positive correlations between *Bacillus* and Avail. P and Exch. K, as well as a negative correlation between *Bacillus* and BD, albeit these correlations were not statistically significant. Contrarily, significant correlations were observed between the genus *Geodermatophillus* and pH (positive) and TN (negative) (see in Supplementary Figure S1).

#### 3.3.2. Taxonomic Distribution in RSC-12Y

Before burning, the most abundant phyla were Proteobacteria (21.57%), Actinobacteria (15.43%), and Acidobacteria (14.17%). Burning significantly changed the abundance of some phyla, including Firmicutes, Proteobacteria, Acidobacteria, Planctomycetes, and Verrucomicrobia. The abundance of Firmicutes increased from 6% to 61% after burning (5 min), then decreased to 16% at 3 months (rainy season) and 8% after 12 months (summer). Proteobacteria decreased from 22% to 8% after burning (5 min), then increased to 22% after 3 months and declined to 10% after 12 months. Similarly, Acidobacteria also decreased from 14% to 4% after burning and increased to 7% after 12 months. However, Actinobacteria did not change significantly immediately after burning (Figure 3b).

**Figure 5:**
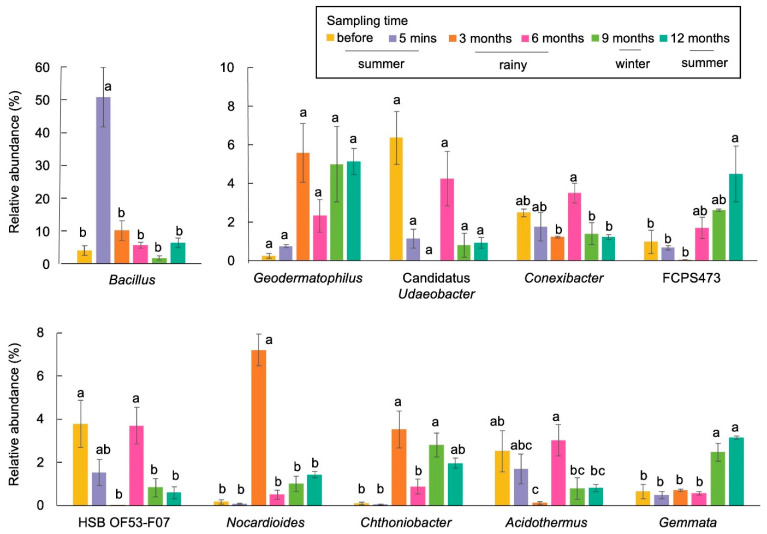
Bar plot showing the abundance of the most abundant genera in RSC-12Y. Different letters in the plots indicate statistically different (*p* < 0.05).

At the genus level, *Bacillus* (1.43%), Candidatus *Udaeobacter* (1.37%), and HSB OF53-F07 (1.49%) were the most prevalent taxa in samples before burning. After burning (5 min), the abundance of *Bacillus* increased significantly, whereas that of *Chthoniobacter* decreased (Figure 5). There were some taxa which increased significantly during the wet season and decreased during the dry season. These included *Conexibacter*, HSB OF53-F07, and *Acidothemus*. One year after burning, the most prevalent taxa belonged to FCPS473 (1.44%), *Bacillus* (1.43%), and *Geodermatophilus* (0.67%).

Spearman’s rank correlation analysis showed positive correlations between Avail. P, Exch. K, Exch. Mg, and Exch. Ca with *Bacillus*, while these nutrients showed negative correlations with other genera. Soil BD and clay showed negative correlations with *Bacillus* but positive with *Geodermatophillus*, FCPS473, *Chthoniobacter*, and *Gemmata*. However, no significant differences were observed in any of these correlations (see in Supplementary Figure S2).

### 3.4. Community Composition and Correlation with Soil Properties

**Figure 6:**
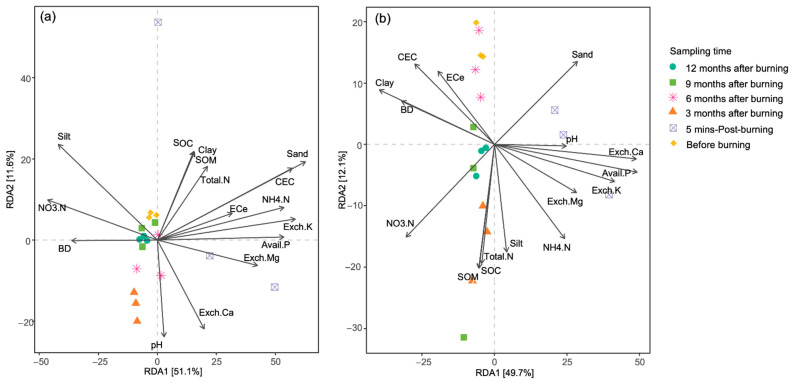
RDA ordination illustrating soil properties significantly correlated with bacterial community composition in (**a**) RSC-6Y and (**b**) RSC-12Y. Significant parameters indicated with the Mantel test (*p* < 0.05).

Two-way PERMANOVA analysis revealed that both sampling time and site significantly affected beta diversity or bacterial community composition (Table 4). Bacteria in each time point and site were separate from each other, and this indicated the difference in the community compositions (see in Supplementary Figure S3). One year after burning, the community compositions of bacteria were not similar to those before burning. Thus, redundancy analyses (RDAs) in the two study sites were performed separately. RDA showed that soil properties explained 62.7% and 61.8% of the total variations in the community compositions in RSC-6Y and RSC-12Y, respectively (Figure 6). According to the Mantel test, several soil parameters (i.e., pH, SOM, SOC, ECe, CEC, NO_3_-N, Avail. P, Exch. K, TN, BD, sand, and silt) significantly impacted the bacterial communities. Specifically, pH and NO_3_-N were significant only in RSC-12Y (Figure 6, Table 5).

### 3.5. Predictive Function

**Figure 7:**
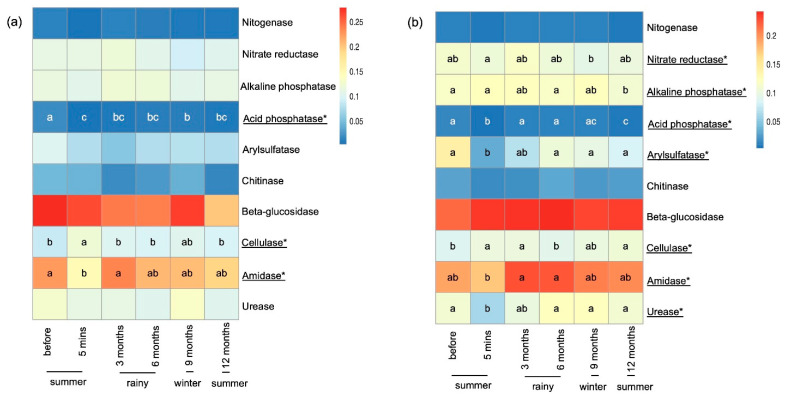
The abundance of predictive enzymes in RSC-6Y (**a**) and RSC-12Y (**b**). Color represents relative abundance of each enzyme. * denote statistically significant (*p* < 0.05).

A total of 19,265 (99.85%) ASVs were assessed for their impact on various soil functions. Specifically, 10 enzymes were highlighted in relation to soil systems, including nitrogenase, nitrate reductase, alkaline phosphatase, acid phosphatase, arylsulfatase, chitinase, β-glucosidase, cellulase, amidase, and urease. In both study sites, acid phosphatase levels decreased significantly immediately after burning and increased after a few months. Similar trends were observed for nitrate reductase and urease in the RSC-12Y site. In contrast, cellulase and amidase levels in both study sites increased significantly immediately after burning and then decreased after some months (Figure 7).

**Figure 8:**
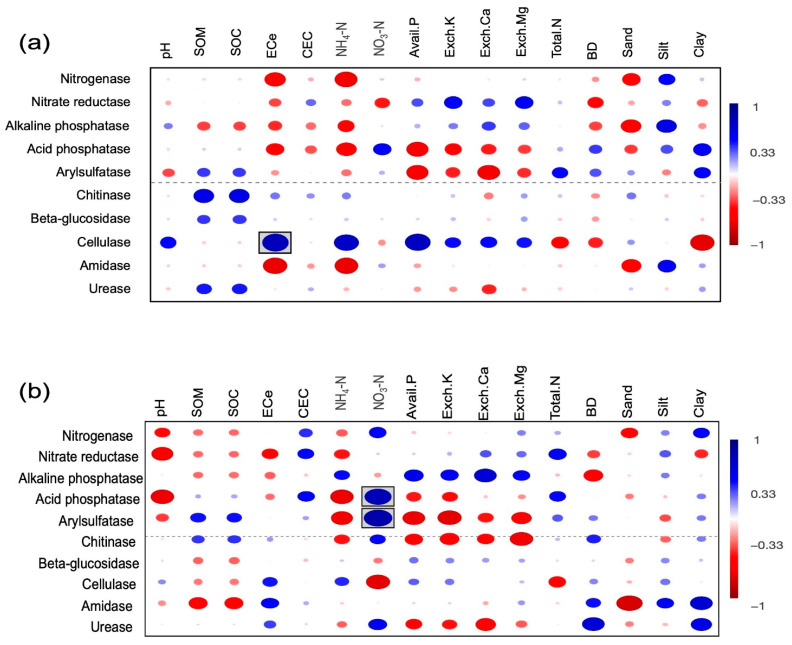
Correlations between the predictive enzymes and soil properties in RSC-6Y (**a**) and RSC-12Y (**b**). Color represents correlation coefficient. Blue = positive correlation, red = negative correlation, and circles within boxes are significant correlations. Circle size corresponds with the correlation coefficient.

In terms of correlations, a significant positive correlation was observed between cellulase and ECe (electrical conductivity of the saturation extract) in RSC-6Y (Figure 8a). In the case of RSC-12Y, significant positive correlations were observed between acid phosphatase and arysulfatase as well as between acid phosphatase and NO_3_-N (nitrate nitrogen) (Figure 8b).

## 4. Discussion

### 4.1. Effects of Seasonal Variations on Soil Microbial Communities

Soil microbial composition changes with the seasons, influenced by variations in soil temperature, moisture, and nutrients content [49,50,51]. Furthermore, seasonal variations lead to changes in environmental conditions, including photosynthesis, root exudates, and the accumulation of litter, which can have an impact on soil microbial communities [52,53]. The data in Table 4 showed that sampling time significantly impacted all diversity indices. Alpha diversity indices across all samples showed a decline immediately after burning and increased with the onset of the early rainy season until the summer season (Figure 2). This trend may be attributed to the increase in soil moisture resulting from precipitation during the rainy season, along with the release of available nutrients from ash and charcoal following the fire (Table 2 and Table 3). Additionally, the increase in temperature from late winter to the summer season contributes to an increase in the composition of soil bacterial communities. The data presented herein are in accord with those reported by Luo et al. [54], who also demonstrated that the microbial community in an orchard in southeastern China exhibited a peak during the summer, likely attributed to the increases in temperature and rainfall. Similar trends have also been observed in diverse ecosystems, including an evergreen broadleaf forest in southwestern China [55], temperate grasslands [56], an apple orchard in the temperate monsoon climate zone [57], and the Mongolian oak-dominant Gwangneung forest in Korea [58].

Seasonal changes in climate parameters can have a significant impact on the growth of vegetation, which directly affects the microbial community [59,60]. This trend may be due to the variations in the decomposition rate of litter [61] and substances released through root exudates [62]. It is also in accord with the trend observed in the alpha diversity indices shown in Figure 2. Soil moisture content plays a crucial role in plant growth and the microbial community by influencing osmotic potential, nutrient and energy transport, and cellular metabolism [63]. Griffths et al. [64] observed that increased availability of carbon sources and root growth during the growing season led to an increase in soil microbial biomass and activity. Jefferies et al. [65], Zhang et al. [66], and Li et al. [67] also reported that soil microbial growth was limited during winter but increased during summer due to an increase in temperature.

In RSC fields, fire is an anthropogenic disturbance that significantly alters soil microbial communities (Figure 1 and Figure 2), which play a crucial role in supporting the recovery process following biomass burning, particularly in response to seasonal variations (Figure 3, Figure 4 and Figure 5). The abundance of Firmicutes, Proteobacteria, Acidobacteria, and Planctomycetes increased after the occurrence of fire. After the fire, there was also a significant increase in the abundance of Firmicutes, making it the most prominent group. However, the abundance of Actinobacteria remained unchanged immediately after burning and exhibited variation throughout the seasons. It showed an increase during the rainy season but a decrease during the winter and summer seasons, which could be attributed to intense competition and interactions between microbial species (Figure 3). The phylum Firmicutes includes diverse thermophilic, antibiotic-producing, and endospore-forming organisms that exhibit high resistance to desiccation, heat, and radiation [68]. A study by Nelson et al. [69] reported that Actinobacteria possess genes associated with heat resistance, rapid growth, and the ability to utilize pyrogenic carbon, which enables their survival following a fire. At the genus level, the abundance of *Sphingomonas* decreased significantly after the fire event in RSC-6Y. However, its abundance showed a significant increase during the rainy season and decreased during the dry season (winter and summer) (Figure 4). This trend suggests that *Sphingomonas* may be a species that exhibits a rapid response to changes in precipitation and temperature, possibly indicating its adaptability to fluctuating environmental conditions. In the RSC-12Y site, the genera *Conexibacter*, HSB OF53-F07, and *Acidothemus* exhibited a positive response during the rainy season and a decrease during the dry season (Figure 4). In contrast to the other genera, the abundance of FCPS473 and *Gemmata* remained unchanged after the occurrence of fire and exhibited a significant increase during the dry season (Figure 4 and Figure 5).

### 4.2. Relationship between Soil Physical and Chemical Properties, and Soil Bacterial Communities

The combustion of biomass and SOM during the fire resulted in the accumulation of ash on the soil surface. This ash accumulation plays a crucial role in altering the physical and chemical properties of the soil [70], ultimately influencing the dynamics of soil microbial communities [71]. The occurrence of fire significantly increased the soil pH, ECe, NH_4_-N, and nutrients (Avail. P, Exch. K, Exch. Ca, and Exch. Mg) (Table 2 and Table 3). This increase was attributed to the deposition of ash during the fire event. However, over time, these values declined due to runoff, leaching, and the movement of nutrients into deeper soil layers through infiltration and percolation. These findings are also in accord with those reported by previous studies [3,12,19,72,73,74,75].

Fire behavior directly impacts the extent of combustion and the maximum temperature reached during the fire event, which in turn influence the soil’s physical, chemical, and biological properties. High-intensity fires result in the high volatilization of SOM and oxidation processes in the topsoil layer [76]. On the other hand, incomplete combustion during low-intensity fires can produce semi-pyrolyzed ash [77]. The accumulation of ash affects soil microbial communities by increasing nutrient availability in the short term [73]. Additionally, the darkening of the soil due to ash deposition reduces the albedo, resulting in an increase in soil surface temperature [78]. The data presented herein show significant correlations between bacterial communities and several soil properties, including pH, SOM, SOC, ECe, CEC, NO_3_-N, Avail. P, Exch. K, TN, BD, and the contents of sand and silt (Table 5). The data presented herein also indicate an increase NO_3_-N and a decrease in NH_4_-N during the rainy and winter seasons (3, 6, and 9 months after burning, corresponding to June, September, and December) (Table 3). This observation suggests a rapid growth of soil microorganisms in the tropical zone, which can be attributed to the high soil moisture conditions during these seasons. These findings are also consistent with the trend observed in the alpha diversity indices (Figure 2), implying that soil bacteria, particularly *Bacillus*, play a role in the post-fire ecosystem by oxidizing NH_4_-N to NO_2_-N and subsequently converting it to NO_3_-N, which is important for plant growth. A study conducted in northern Sweden by DeLuca et al. [79] reported findings similar to those presented herein. The data in Table 5 specifically show that pH and NO_3_-N played a significant role in changing the bacterial communities in RSC-12Y, where fire intensity and soil temperature were higher compared to RSC-6Y (Table 1). These findings may be attributed to the significant impact of increased soil temperature and soil pH on nitrogen cycle processes. Jones et al. [80] and Verma and Jayakumar [81] have revealed that an increase in soil temperature and higher pH have an impact on the dynamics of inorganic nitrogen (NH_4_-N and NO_3_-N) through mineralization and nitrification processes, as well as the ammonification process [12].

The short-term increase in available nutrients and soil pH following a fire can facilitate the rapid regrowth of plants, leading to an increased release of plant root exudates. These exudates play a crucial role in changing the composition of soil bacteria [82]. Meharg and Killham [83] and Ren et al. [84] observed that a high pH value promotes bacterial growth by increasing the exudation of organic compounds from plants. This provides another possible explanation for the observed increase in bacterial richness and diversity three months after the fire in our study (Figure 2). Fire alters the functional diversity of microbial communities, which is essential for the recovery of ecosystem processes, influencing decomposition and nutrient cycling [85]. D’Ascoli et al. [86] observed short-term changes in functional diversity after fire in the Mediterranean region, suggesting rapid recovery despite persistent reductions in microbial community activity and structural changes. Fontúrbel et al. [71] observed higher values of diversity indices and pH immediately after fire, indicating more favorable soil conditions for stimulating bacterial populations. However, fire-induced changes in the functional diversity of microbial communities in RSC fields need to be further studied in the future.

## 5. Conclusions

Understanding the impact of fire on soil physicochemical properties and bacterial communities in RSC fields is crucial for effective post-fire management and regeneration of burnt areas. The data presented herein show that immediately after the fire, there was a decrease in soil bacterial community diversity and an increase in soil nutrient levels at the 0–2 cm soil depth. However, 3 months after burning (during the rainy season), there was a recovery in the abundance of the soil bacterial communities, despite the decline in soil nutrient availability. The composition of soil microbial communities was influenced by seasonal variation and the soil’s physicochemical properties. After one year of burning, there was an increase in soil nutrient levels, pH, and ECe compared to those of the initial values (pre-burning), along with an increase in bacterial richness. However, the diversity of the bacterial communities reverted to pre-burning levels.

## Figures and Tables

**Table 1:** Soil moisture, soil temperature, and fire behaviors of study sites (mean ± SD).

Sites	Variables	Time Points						Fire Temperature in the Litter Top (°C) (min–max)	Flame Length (m)	Flame Residence Time (s)	Spread Rate (m/min)
		Pre-Burning	5 min after Burning	3 Months after Burning	6 Months after Burning	9 Months after Burning	12 Months after Burning				
RSC-6Y	Soil moisture (%)	34.2 ± 5.5 ^b^	31.7 ± 6.9 ^b^	41.3 ± 7.5 ^a^	43.5 ± 8.0 ^a^	33.8 ± 5.5 ^b^	32.5 ± 5.5 ^b^	253–612	3.6 ± 1.0	22.0 ± 19.0	12.5 ± 8.5
	Soil temperature (°C)	23.3 ± 3.1 ^a^	46.0 ± 2.5 ^b^	25.2 ± 3.5 ^a^	23.4 ± 3.0 ^a^	26.5 ± 3.0 ^a^	27.5 ± 2.5 ^a^				
RSC-12Y	Soil moisture (%)	35.8 ± 4.4 ^b^	30.4 ± 5.8 ^b^	40.1 ± 6.0 ^a^	45.4 ± 5.8 ^a^	32.5 ± 6.0 ^b^	32.1 ± 6.2 ^b^	315–754	5.5 ± 2.0	33.0 ± 21.0	15.5 ± 9.0
	Soil temperature (°C)	23.6 ± 2.2 ^a^	47.8 ± 1.0 ^b^	25.1 ± 2.0 ^a^	23.5 ± 2.5 ^a^	26.1 ± 3.5 ^a^	27.5 ± 3.0 ^a^				

^a,b^ significant statistical differences (*p* < 0.05), as determined by using one-way repeated measures ANOVA with post-hoc Tukey’s HSD.

**Table 2:** Soil physical and chemical properties: bulk density (BD), electrical conductivity (ECe), soil organic matter (SOM), soil organic carbon (SOC), total nitrogen (TN), and proportion of sand, silt and clay.

Site	Time Point	pH (1:1)		EC_e_ (dS m^−1^)		BD (Mg m^−3^)		SOM (%)		SOC (%)		TN (%)		%Sand		%Silt		%Clay	
		Mean	SD	Mean	SD	Mean	SD	Mean	SD	Mean	SD	Mean	SD	Mean	SD	Mean	SD	Mean	SD
RSC-6Y	pre-burning	5.69 ^a^	0.03	0.14 ^a^	0.01	1.35 ^a^	0.02	7.03 ^a^	0.31	4.08 ^a^	0.18	0.26 ^a^	0.02	24.18 ^a^	0.71	48.30 ^a^	0.57	27.52 ^a^	0.74
	5 min after burning	6.72 ^b^	0.05	1.22 ^b^	0.01	1.29 ^a^	0.01	5.74 ^b^	0.11	3.33 ^b^	0.06	0.19 ^b^	0.01	34.46 ^b^	0.48	45.47 ^a^	0.04	20.07 ^b^	0.52
	3 months after burning	6.93 ^b^	0.13	1.60 ^b^	0.01	1.31 ^a^	0.02	5.21 ^b^	0.11	3.02 ^b^	0.06	0.14 ^b^	0.01	22.16 ^a^	0.05	54.12 ^b^	0.05	23.72 ^b^	0.09
	6 months after burning	6.97 ^b^	0.12	1.11 ^b^	0.02	1.32 ^a^	0.01	5.33 ^b^	0.09	3.09 ^b^	0.06	0.19 ^b^	0.02	22.38 ^a^	0.05	53.04 ^b^	0.05	24.58 ^a^	0.09
	9 months after burning	7.02 ^b^	0.11	1.14 ^b^	0.01	1.39 ^a^	0.01	5.38 ^b^	0.06	3.12 ^b^	0.04	0.13 ^b^	0.01	22.83 ^a^	0.05	50.91 ^a^	0.04	26.26 ^a^	0.08
	12 months after burning	6.37 ^b^	0.15	1.11 ^b^	0.01	1.42 ^a^	0.01	5.40 ^b^	0.02	3.13 ^b^	0.01	0.21 ^a^	0.02	27.06 ^a^	1.09	45.62 ^a^	0.75	27.32 ^a^	0.84
RSC-12Y	pre-burning	5.31 ^a^	0.03	0.13 ^a^	0.01	1.28 ^a^	0.02	7.45 ^a^	0.21	4.32 ^a^	0.12	0.31 ^a^	0.02	35.19 ^a^	0.90	45.66 ^a^	1.14	19.15 ^a^	1.99
	5 min after burning	6.26 ^b^	0.03	0.56 ^b^	0.02	1.25 ^a^	0.01	6.14 ^b^	0.21	3.56 ^a^	0.12	0.21 ^b^	0.03	33.43 ^a^	2.77	51.29 ^b^	0.36	15.28 ^a^	3.00
	3 months after burning	6.23 ^b^	0.02	0.67 ^b^	0.01	1.27 ^a^	0.02	5.48 ^b^	0.08	3.18 ^a^	0.05	0.23 ^b^	0.02	22.31 ^b^	0.16	54.19 ^b^	0.09	23.50 ^b^	0.18
	6 months after burning	6.03 ^b^	0.07	1.03 ^b^	0.07	1.28 ^a^	0.02	6.03 ^b^	0.07	3.50 ^a^	0.04	0.24 ^b^	0.02	22.53 ^b^	0.16	53.10 ^b^	0.08	24.37 ^b^	0.18
	9 months after burning	6.12 ^b^	0.03	1.20 ^b^	0.03	1.35 ^a^	0.02	6.22 ^b^	0.09	3.61 ^a^	0.05	0.16 ^b^	0.02	22.98 ^b^	0.17	50.98 ^b^	0.08	26.04 ^b^	0.19
	12 months after burning	6.04 ^b^	0.09	1.20 ^b^	0.06	1.36 ^a^	0.02	6.28 ^b^	0.11	3.64 ^a^	0.06	0.20 ^b^	0.02	26.70 ^b^	0.99	50.64 ^b^	2.35	22.66 ^b^	1.53

^a,b^ significant statistical differences (*p* < 0.05), as determined by using one-way repeated measures ANOVA with post-hoc Tukey’s HSD.

**Table 3:** Soil chemical properties: cation exchange capacity (CEC); available phosphorus (avail.P); exchangeable K, Ca, and Mg, NH4-N, and NO3-N.

Site	Time Point	CEC (cmol kg^−1^)		Avail. P (mg kg^−1^)		Exch. K (mg kg^−1^)		Exch. Ca (mg kg^−1^)		Exch. Mg (mg kg^−1^)		NH_4_-N (mg kg^−1^)		NO_3_-N (mg kg^−1^)	
		Mean	SD	Mean	SD	Mean	SD	Mean	SD	Mean	SD	Mean	SD	Mean	SD
RSC-6Y	pre-burning	12.52 ^a^	0.00	2.49 ^a^	0.09	147.66 ^a^	3.38	455.21 ^a^	16.26	175.99 ^a^	4.40	7.11 ^a^	0.00	14.21 ^a^	0.00
	5 min after burning	13.99 ^a^	0.36	50.31 ^b^	1.92	398.92 ^b^	9.72	1024.87 ^b^	46.60	278.51 ^b^	9.27	68.68 ^b^	4.10	0.00 ^b^	0.00
	3 months after burning	11.27 ^a^	0.00	30.75 ^b^	0.37	256.20 ^b^	7.70	1244.45 ^b^	70.04	265.38 ^b^	5.77	28.42 ^c^	0.00	14.21 ^a^	0.00
	6 months after burning	10.39 ^a^	0.07	25.82 ^b^	0.31	215.20 ^b^	6.47	1045.33 ^b^	58.83	222.92 ^b^	4.84	35.53 ^c^	0.00	28.42 ^b^	0.00
	9 months after burning	10.72 ^a^	0.37	16.52 ^c^	0.20	137.72 ^a^	4.14	669.01 ^a^	37.65	142.66 ^a^	3.10	48.35 ^c^	0.00	35.53 ^b^	0.00
	12 months after burning	11.68 ^a^	0.16	11.01 ^c^	0.29	135.38 ^a^	23.61	634.73 ^a^	23.90	139.54 ^a^	10.51	35.53 ^c^	0.00	14.21 ^a^	0.00
RSC-12Y	pre-burning	12.52 ^a^	0.00	2.62 ^a^	0.12	93.94 ^a^	1.02	174.54 ^a^	6.77	87.43 ^a^	0.60	35.53 ^a^	0.00	132.63 ^a^	4.10
	5 min after burning	10.65 ^a^	0.63	50.31 ^b^	6.74	334.23 ^b^	4.93	546.43 ^b^	11.81	165.03 ^b^	8.39	90.00 ^b^	4.10	7.11 ^b^	0.00
	3 months after burning	14.40 ^a^	0.00	19.79 ^c^	0.91	248.57 ^b^	2.45	311.13 ^b^	11.51	174.38 ^b^	10.82	78.16 ^c^	0.00	35.53 ^c^	0.00
	6 months after burning	11.26 ^a^	0.33	16.62 ^c^	0.76	208.80 ^b^	2.06	261.34 ^b^	9.67	146.47 ^b^	9.09	78.16 ^c^	0.00	59.21 ^d^	4.10
	9 months after burning	11.92 ^a^	0.42	10.63 ^c^	0.49	133.63 ^a^	1.32	167.25 ^a^	6.18	93.73 ^a^	5.82	78.72 ^c^	0.15	60.89 ^d^	0.19
	12 months after burning	12.30 ^a^	0.15	8.39 ^c^	0.27	106.27 ^a^	6.63	139.66 ^a^	1.40	83.16 ^a^	8.17	78.16 ^c^	0.00	35.53 ^c^	0.00

^a–d^ significant statistical differences (*p* < 0.05), as determined by using one-way repeated measures ANOVA with post-hoc Tukey’s HSD.

**Table 4:** Alpha and Beta diversity indices of bacteria.

Factors	Alpha Diversity				Beta Diversity
	Richness		Diversity Index		Bray–Curtis
	Observed	ACE	Shannon	Simpson	
Site	0.44	0.54	0.13	0.41	3.10 *
Sampling time	14.28 *	14.03 *	15.98 *	7.96 *	4.10 *
Site:Sampling time	0.22	0.17	0.13	0.3	2.16 *

* denote statistically significant (*p* < 0.05), as determined by using two-way ANOVA and PERMANOVA analysis.

**Table 5:** Correlation coefficient between bacterial community and soil properties.

Soil Properties	RSC-6Y		RSC-12Y	
	Correlation Coefficient	*p*-Value	Correlation Coefficient	*p*-Value
pH	0.184	0.054	0.257	0.008 *
SOM	0.339	0.004 *	0.302	0.009 *
SOC	0.341	0.004 *	0.300	0.010 *
ECe	0.296	0.005 *	0.404	0.001 *
CEC	0.354	0.002 *	0.298	0.003 *
NH_4_-N	0.410	0.001 *	0.304	0.001 *
NO_3_-N	0.131	0.119	0.442	0.001 *
Avail. P	0.424	0.001 *	0.320	0.002 *
Exch. K	0.340	0.001 *	0.303	0.003 *
Exch. Ca	0.235	0.013 *	0.296	0.006 *
Exch. Mg	0.183	0.030 *	0.218	0.026 *
Total. N	0.138	0.100	0.155	0.086
BD	0.237	0.012 *	0.228	0.012 *
Sand	0.301	0.011 *	0.360	0.001 *
Silt	0.218	0.019 *	0.221	0.034 *
Clay	0.333	0.004 *	0.255	0.023 *

* denote statistically significant (*p* < 0.05), as determined by using Mantel test.

## Data Availability

The original contributions presented in the study are publicly available. The data can be found at the National Center for Biotechnology Information (NCBI) under the BioProject accession number PRJNA1003674.

## Supplementary Materials

The following supporting information can be downloaded at: https://www.mdpi.com/article/10.3390/biology13060383/s1. Figure S1: Correlation between the abundant genera and soil properties in RSC-6Y. Color represents correlation coefficient. Blue = positive correlation, red = negative correlation, and circles within boxes are significant correlations; Figure S2: Correlation between the abundant genera and soil properties in RSC-12Y. Color represents correlation coefficient. Blue = positive correlation, red = negative correlation, and circles within boxes are significant correlations; Figure S3: NMDS ordination based on Bray–Curtis distances showing bacterial community compositions across all samples.
