# Supplementary material for: Fire-Induced Changes in Soil Properties and Bacterial Communities in Rotational Shifting Cultivation Fields in Northern Thailand
Source: Biology (Basel). 2024 May 27;13(6):383. doi: 10.3390/biology13060383 (PMC11200764; doi:10.3390/biology13060383)
Supplement: Supplementary file 1 [file biology-13-00383-s001.zip › biology-3002249-supplementary.pdf]

Supplementary material

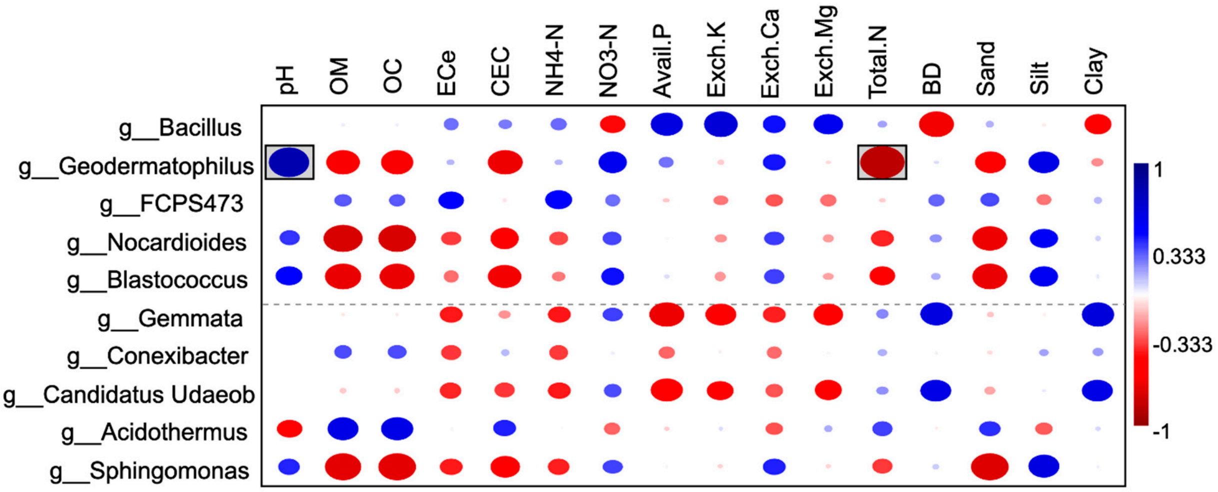

Figure S1: Correlation between the abundance genus and soil properties in RSC-6Y. Color represents correlation coefficient. Blue = positive correlation, Red = negative correlation, circle with Box is the significant correlation.

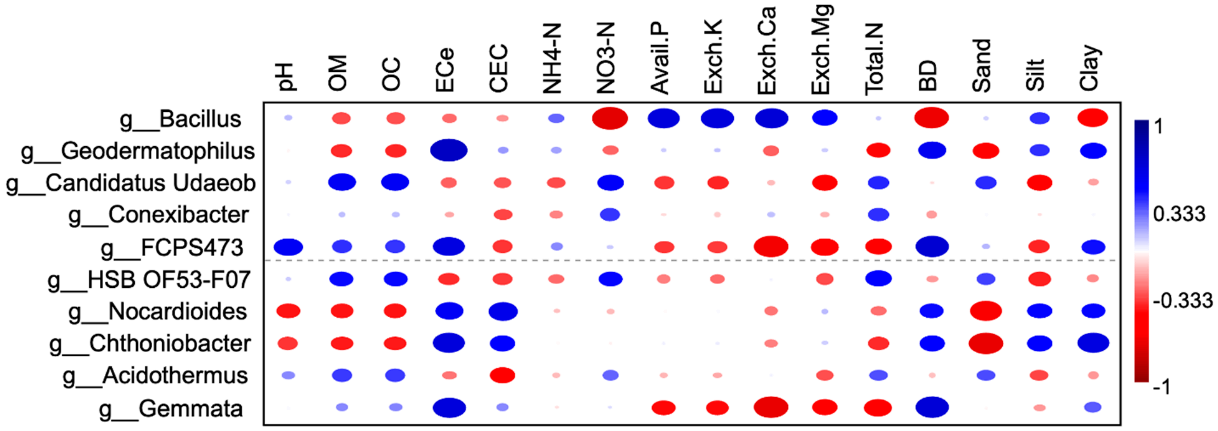

Figure S2: Correlation between the abundance genus and soil properties in RSC-12Y. Color represents correlation coefficient. Blue = positive correlation, Red = negative correlation, circle with Box is the significant correlation.

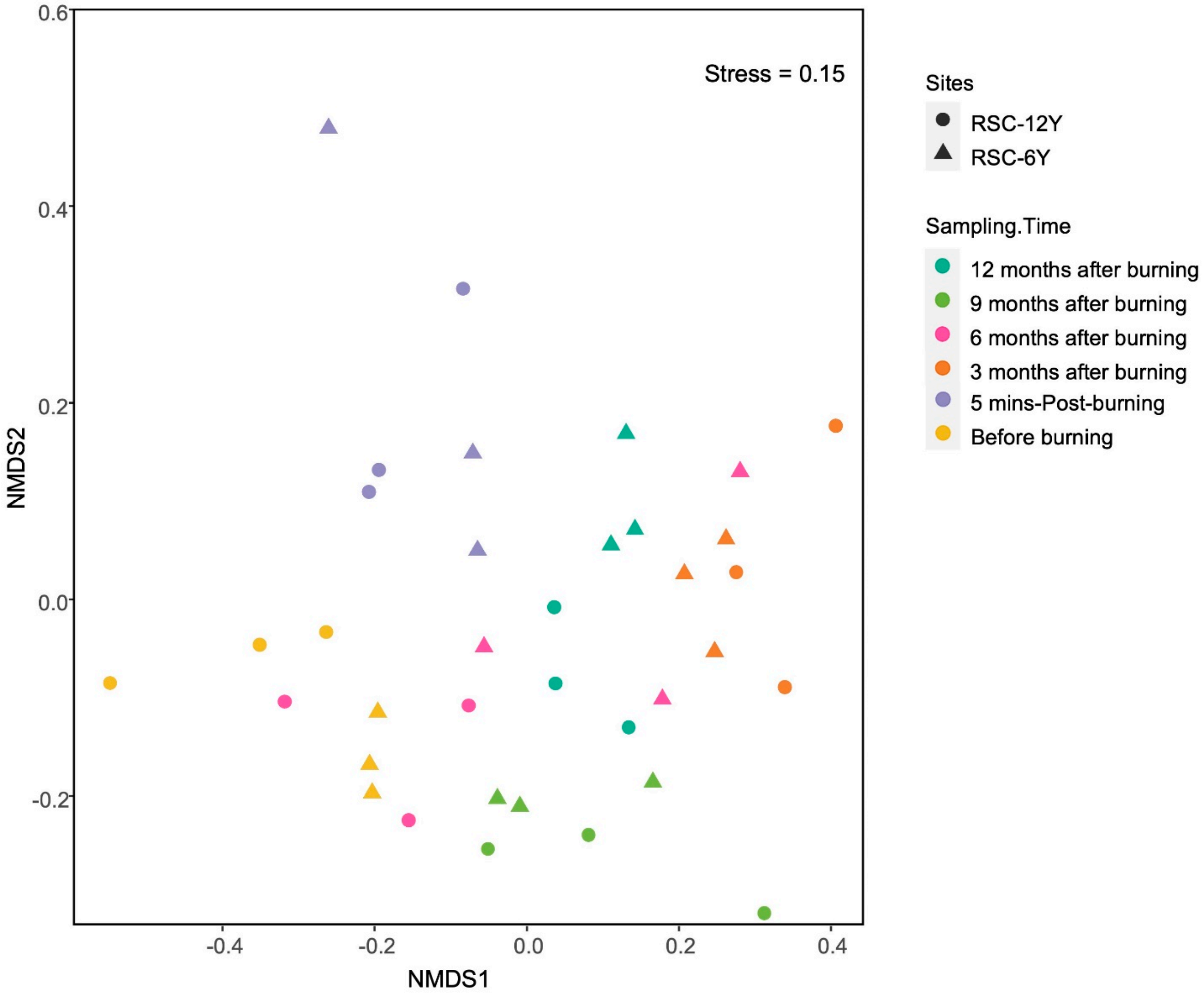

Figure S3: NMDS ordination based on Bray‒ Curtis distance showing bacterial community composition across all samples
